# The incidence and body site of skin cancers in the population groups of Astana, Kazakhstan

**DOI:** 10.1002/hsr2.51

**Published:** 2018-05-31

**Authors:** Pauline McLoone, Philip McLoone, Khalel Imanbayev, Mary Norval

**Affiliations:** ^1^ Nazarbayev University School of Medicine Astana Kazakhstan; ^2^ Institute of Health and Wellbeing University of Glasgow Scotland UK; ^3^ Department of Oncology Astana Medical University Astana Kazakhstan; ^4^ Biomedical Sciences University of Edinburgh Edinburgh Scotland UK

**Keywords:** Astana, basal cell carcinoma, cutaneous malignant melanoma, incidence, Kazakhstan, squamous cell carcinoma of the skin

## Abstract

**Background and aims:**

Data on cutaneous malignant melanoma (CMM), squamous cell carcinoma of the skin (SCC), and basal cell carcinoma (BCC) in populations consisting of multi‐racial groups in the Commonwealth of Independent States are limited. Here, the main aim was to analyse the incidence and body site of these cancers in the population groups of Astana, Kazakhstan (2007‐2016).

**Methods:**

Annual age standardised incidences and body sites of BCC, SCC, and CMM in Astana's population, divided into “Kazakhs and other Turkic/Asian” and “Russian and other European/Caucasian” groups, were calculated from histologically confirmed cases reported to Astana Oncology Centre.

**Results:**

During the period January 2007 to October 2016, 647 skin cancers were diagnosed. The age and sex standardised incidence of BCC, SCC, and CMM increased significantly between 2007 to 2011 and 2012 to 2016. Higher incidences occurred in the Russian and other European/Caucasian group compared with the Kazakh and other Turkic/Asian group for the 3 skin cancers. BCC was the most common type of skin tumour, followed by SCC, and then CMM, in both population groups and sexes. The head/neck was the commonest site for BCC and SCC in all groups. For CMM, the most frequent site was the trunk in the Russian group and the head/neck in the Kazakh group.

**Conclusion:**

The incidence of skin tumours in Astana rose over the past 10 years. Differences in skin phototypes and sun exposure/ protection behaviours may account for the more frequent occurrence of skin tumours in the Russian population group compared with the Kazakh population group.

## 1 INTRODUCTION

Information on the incidence of the most common skin cancers,cutaneous malignant melanoma (CMM), squamous cell carcinoma of the skin (SCC), and basal cell carcinoma (BCC), is available for many Western countries^1^, ^2^, ^3^ but is limited for Commonwealth of Independent States (former Soviet Republics) such as Kazakhstan. This is particularly true for locations where the population is multi‐ethnic, containing a range of skin phototypes.

Kazakhstan is a former Soviet Republic that became independent following the dissolution of the Soviet Union in 1991. Its capital city was transferred to Astana in 1997. At the time of the 2009 census, Astana's population comprised 89.1% Kazakhs, 4.5% Russians, 0.9% Ukrainians, and 2.4% Uzbeks.^4^ Since 2009, Astana's population increased by around 50%, and was 872 619 in 2016,^5^ consisting of 75.5% Kazakhs, 15.3% Russians, 1.6% Ukrainians, 1.1% Uzbeks, 1% Germans, and several minority groups.^6^ Astana has an extreme continental climate, with warm summers and long, cold winters. It can be as hot as +35°C in the summer, while in the winter temperatures as low as −35°C are not uncommon. The UV index ranges from 1 in the winter to 7 in July/August, and daily hours of sunshine vary from 3 in November/December to 11 in June/July.^7^ Astana is located in a flat steppe region, encompassing 722 km^2^, and lies at latitude 51.6^0^N, longitude 71.5°E, and altitude 347 m.

Data from the Ministry of Health in Kazakhstan (2015) indicate that skin cancer is one of the most common forms of cancer in the country,^8^ but a detailed analysis relating to the population groups in Kazakhstan has not previously been reported. In this study, the incidence and body sites of BCC, SCC, and CMM in the multi‐national populations of Astana were investigated.

## 2 METHODS

Information on histologically confirmed cases of CMM, BCC, and SCC, diagnosed in Astana between the years 2007 and 2016, was obtained from the Astana Oncology Centre. The Astana Oncology Centre is a cancer diagnostic and treatment centre where all histologically confirmed skin cancer cases are accurately registered in a central oncology register. The centre serves the population of Astana and treats inpatients as well as outpatients. The Institutional Research Ethics Committee of Nazarbayev University granted institutional review board exemption for the study, based on the fact that the study did not involve the participation of human subjects, and that only de‐identified histologically confirmed skin cancer cases from the Astana Oncology Centre cancer registry would be used for the analysis. Furthermore, patients diagnosed with skin cancer at the Astana Oncology Centre were informed that their data could be used for research. The type of skin cancer, age at diagnosis, sex, nationality, cancer stage, body site, and date of diagnosis were recorded. Nationality, a parameter commonly recorded in hospitals and clinics in Kazakhstan, was determined from passports or identity cards. Counts of the population of Astana by age, sex, and nationality were obtained from the 2009 population census.^4^ Mid‐year population estimates for 2017 by age and sex, along with estimated proportions of national groups, were obtained from the Ministry of National Economy of the Republic of Kazakhstan, Committee on Statistics (2017).

Across and within the different population groups of Astana, there is a considerable range of nationalities and skin phototypes. For the purposes of this study, we classified Russians, Ukrainians, Germans, Belarusians, Chechens, and Poles as European/Caucasian skin types, and Kazakhs, Tatars, Uzbeks, Koreans, Azerbaijanis, Turks, Bashkirs, Uyghurs, Dungans, and Tajiks as Turkic/Asian skin types. The numbers of skin cancers in the Kazakh and Russian populations were assessed separately and then combined with other Turkic/Asian and other European/Caucasian, respectively, for calculation of incidence and percentage of specific cancer body site location, because the numbers in the minority groups were small. The percentage of SCC, BCC, and CMM reported to occur on the head/neck, trunk, upper extremity, and lower extremity was calculated for the years 2007 to 2016 in the 2 groups: Kazakh and other Turkic/Asian, and Russian, and other European/Caucasian.

For all skin cancers and each type, direct age standardised (ages 20+) incidence rates expressed per 100 000 population with 95% confidence intervals were produced for all of Astana's population for the grouped years 2007 to 2011 and 2012 to 2016. For the period 2007 to 2011, the population denominators were taken from the 2009 population Census. For the period 2012 to 2016, the denominator for rates were taken from interpolated 2014 mid‐year population estimates. We did not use different nationality counts for this period. The reference population for the age standardisation was the European standard population.^9^ For the period 2007 to 2011, age standardised rates were calculated for the population grouped into Russian and other European/Caucasian, and Kazakh and other Turkic/Asian. Age standardised rates for the period 2012 to 2016 for the population grouped into Russian and other European/Caucasian and Kazakh and other Turkic/Asian were not produced because counts of the population divided by age, sex, and ethnic group were not available for the inter‐census years 2012 to 2016.

Differences in categorical demographic and clinical characteristics of patients by cancer type were assessed using Pearson chi‐square test or Monte Carlo permutation tests of independence, when appropriate. Kruskal‐Wallis test was used to test for differences in median age. All tests were 2‐sided, and the standard significance level of 0.05 was used throughout. Statistical analyses were performed using Stata v14.0.

## 3 RESULTS

Between January 2007 and October 2016, 647 skin cancers were diagnosed. The age of the patients at diagnosis ranged from 10 to 91 years (median 66, IQR 56‐73). Table 1 shows that 58.9% of cancers were found in women and 41.1% in men, with the majority in the Russian population (46.7%), followed by the Kazakh population (26.6%). The most common cancer type was BCC (67.9%), followed by SCC (15.3%), and then CMM (11.9%). The most frequent body site was the head/neck (73%), followed by the trunk (12.1%), the lower extremity (7.1%), and the upper extremity (4.3%). The majority of the skin cancers were stage I (76%).

**Table 1:** Preliminary analysis of skin cancer cases recorded at Astana Oncology Centre 2007 to 2016

Characteristics		*N*	(%)
Sex	Men	266	(41.1)
	Women	381	(58.9)
Nationality	Russian	302	(46.7)
	Kazakh	172	(26.6)
	Other European/Caucasian	133	(20.6)
	Other Turkic/Asian	38	(5.9)
	Not reported	2	(0.3)
Cancer type	SCC	99	(15.3)
	BCC	439	(67.9)
	CMM	77	(11.9)
	Other	32	(5.0)
Stage	I	492	(76.0)
	II	130	(20.1)
	III	14	(2.2)
	IV	4	(0.6)
	Not reported	7	(1.1)
Site	Head and neck	472	(73.0)
	Trunk	78	(12.1)
	Lower extremity	46	(7.1)
	Upper extremity	28	(4.3)
	Not reported	23	(3.6)

### 3.1 Characteristics by cancer type

Table 2 indicates whether there was an association between patient demographic/clinical characteristics and type of cancer. The median age of patients with BCC, SCC, and CMM was 66, 68, and 57 years, respectively. Median age (95% CI) of CMM patients was lower than median age of patients with BCC or SCC (57 (54 to 62) years, 66 (65 to 68) years, 68 (64 to 71) years, respectively; Kruskal‐Wallis test *P* = 0.01). More than 50% of CMM cases were reported as stage II or higher, compared with 25% and 15% of SCCs and BCCs, respectively. Head/neck was the most common site for SCCs and BCCs, but CMM was more uniformly distributed across body sites. A higher proportion of skin cancers were located on the trunk in men compared with women (17.7%, 95%CI [13.2 to 22.8%] vs 8.1% [5.6 to 11.4%], Pearson chi‐square *P* < 0.001), and on the lower extremity in women compared with men (9.4%, 95% CI [6.7 to 12.8%] vs 3.8% [1.8 to 6.8%], Pearson chi‐square *P* = 0.006).

**Table 2:** Characteristics of skin cancer cases

		SCC		BCC		CMM		Other		
Characteristics		*N*	(%)	*N*	(%)	*N*	(%)	*N*	(%)	*P*‐Value
Sex	Men	34	(34.3)	184	(41.9)	32	(41.6)	16	(50.0)	*P* = 0.38
	Women	65	(65.7)	255	(58.1)	45	(58.4)	16	(50.0)	
Median age (IQR)		68	(56–77)	66	(58–73)	57	(46–65)	63	(50.5–73.5)	*P* < 0.001
Nationality	Russian	49	(49.5)	209	(47.6)	34	(44.2)	10	(31.3)	*P* = 0.09
	Kazakh	28	(28.3)	100	(22.8)	28	(36.4)	16	(50.0)	
	Other European/Caucasian	16	(16.2)	102	(23.2)	11	(14.3)	4	(12.5)	
	Other Turkic/Asian	6	(6.1)	26	(5.9)	4	(5.2)	2	(6.3)	
	Not reported	0	(0.0)	2	(0.5)	0	(0.0)	0	(0.0)	
Stage	I	74	(74.7)	369	(84.1)	37	(48.1)	12	(37.5)	*P* < 0.001
	II	23	(23.2)	62	(14.1)	32	(41.6)	13	(40.6)	
	III	2	(2.0)	3	(0.7)	6	(7.8)	3	(9.4)	
	IV	0	(0.0)	2	(0.5)	1	(1.3)	1	(3.1)	
	Not reported	0	(0.0)	3	(0.7)	1	(1.3)	3	(9.4)	
Site	Head and neck	78	(78.8)	362	(82.5)	18	(23.4)	14	(43.8)	*P* < 0.001
	Trunk	6	(6.1)	40	(9.1)	24	(31.2)	8	(25.0)	
	Upper extremity	6	(6.1)	12	(2.7)	8	(10.4)	2	(6.3)	
	Lower extremity	8	(8.1)	12	(2.7)	20	(26.0)	6	(18.8)	
	Not reported	1	(1.0)	13	(3.0)	7	(9.1)	2	(6.3)	

The *P* value for sex by cancer type is from Pearson chi‐square test of heterogeneity. This tests whether frequency counts are distributed identically in men and women. The *P* value for age comes from Kruskal‐Wallis test which tests whether distribution of age is the same for each cancer type. The *P* values for nationality, stage, and site are from tests of heterogeneity. Each tests whether cancer type frequency counts are distributed identically across nationality, identical across stage, and identical across site. A permutation test based on the chi‐square statistic was used.

### 3.2 Body site of CMM, BCC, and SCC in the population groups

The commonest body site for BCCs and SCCs was the head/neck in all population groups and in both males and females. For CMM, the most frequent body site was the head/neck in Kazakh and other Turkic/Asians (34.4%) and the trunk in Russian and other European/Caucasians (35.6%). However, for each cancer, there was no statistically significant association between body site and national group (Table 3).

**Table 3:** The number and percentage (in brackets) of squamous cell carcinoma of the skin (SCC), basal cell carcinoma (BCC), and cutaneous malignant melanoma (CMM) reported to occur on 4 body sites in Kazakh and other Turkic/Asian and European/Caucasian population groups (2007‐2016)

Cancer	National Grouping	Head and Neck		Trunk		Upper Extremity		Lower Extremity		Not Reported		*P*‐Value
SCC	Kazakh and other Turkic/Asian	26	(76.5)	4	(11.8)	1	(2.9)	2	(5.9)	1	(2.9)	0.19
	Russian and other European/Caucasian	52	(80.0)	2	(3.1)	5	(7.7)	6	(9.2)	0	(0.0)	
BCC	Kazakh and other Turkic/Asian	109	(86.5)	6	(4.8)	3	(2.4)	6	(4.8)	2	(1.6)	0.10
	Russian and other European/Caucasian	252	(81.0)	33	(10.6)	9	(2.9)	6	(1.9)	11	(3.5)	
CMM	Kazakh and other Turkic/Asian	11	(34.4)	8	(25.0)	1	(3.1)	10	(31.3)	2	(6.3)	0.12
	Russian and other European/Caucasian	7	(15.6)	16	(35.6)	7	(15.6)	10	(22.2)	5	(11.1)	
Other	Kazakh and other Turkic/Asian	7	(38.9)	5	(27.8)	2	(11.1)	3	(16.7)	1	(5.6)	0.82
	Russian and other European/Caucasian	7	(50.0)	3	(21.4)	0	(0.0)	3	(21.4)	1	(7.1)	

*P*‐values are for Fishers exact test for heterogeneity. This tests whether frequency counts are distributed identically across different populations.

### 3.3 Age standardised incidence of CMM, BCC, and SCC

During the study period, the age and sex standardised incidence rate of skin cancer increased from 22.1 per 100 000 in 2007 to 2011 to 53.1 per 100 000 in 2012 to 2016. This large rise in incidence was seen in all cancer types and in both sexes (Table 4). Men generally had a higher incidence of skin cancer than women, with the exception of SCC in both time periods, and CMM in 2012 to 2016.

**Table 4:** Age standardised incidence of CMM, BCC, and SCC in Astana, Kazakhstan (2007‐2016)

		Age Standardised Incidence Rate per 100 000 (95% CI)			
	Cancer	2007‐2011		2012‐2016	
Men	Skin cancers	23.6	(18.4 to 28.9)	54.9	(47.0 to 62.8)
	SCC	1.9	(0.6 to 3.2)	7.7	(4.8 to 10.7)
	BCC	18.5	(13.7 to 23.4)	37.6	(30.6 to 43.4)
	CMM	2.2	(0.9 to 3.4)	4.6	(2.6 to 6.6)
Women	Skin cancers	20.6	(16.5 to 24.6)	51.2	(45.2 to 57.1)
	SCC	4.6	(2.7 to 6.5)	8.4	(5.9 to 10.9)
	BCC	14.9	(11.4 to 18.5)	33.4	(28.6 to 38.2)
	CMM	0.9	(0.2 to1.5)	5.1	(3.5 to 6.7)

### 3.4 Age standardised incidence of CMM, BCC, and SCC in the Russian and other European/Caucasian and Kazakh and other Turkic/Asian groups

The incidence of skin cancer among the Russian and other European/Caucasian group during 2007 to 2011 was 81.0 per 100 000 for men and 57.3 for women. This was 1321% and 559% greater than that experienced by the Kazakh and other Turkic/Asian group, respectively, in whom the equivalent rates were 5.7 and 8.7 per 100 000 (Table 5). Rates were higher in the Russian and other European/Caucasian group for each cancer type, and the relative differential between European/Caucasian and Turkic/Asian was greater among men compared with women.

**Table 5:** Age standardised incidence of CMM, BCC, and SCC in the Russian and European/Caucasian, and the Kazakh and Turkic/Asian population groups in Astana, Kazakhstan (2007‐2011)

		Age Standardised Incidence Rate per 100 000 (95% CI) (2007‐2011)			
	Cancer	Russian and other European/Caucasian		Kazakh and other Turkic/Asian	
Men	Skin cancers	81.0	(60.0 to 102.0)	5.7	(3.3 to 8.2)
	SCC	6.9	(0.9 to 13.0)	0.4	(0.0 to 0.8)
	BCC	64.2	(45.4 to 82.9)	4.0	(1.6 to 6.3)
	CMM	6.9	(0.9 to 12.9)	1.4	(0.4 to 2.4)
Women	Skin cancers	57.3	(43.6 to 71.1)	8.7	(5.7 to11.7)
	SCC	14.1	(6.7 to 21.5)	3.1	(1.0 to 5.3)
	BCC	38.4	(27.6 to 49.1)	4.8	(2.7 to 7.0)
	CMM	3.6	(0.0 to 7.6)	0.6	(0.0 to 1.1)

## 4 DISCUSSION

This study has shown that there was a higher incidence of skin cancer in the Russian and other Caucasian/European group than in the Kazakh and other Turkic/Asian group in Astana. In addition, a substantial increase in the incidence rate of skin cancer in both men and women occurred between 2007 to 2011 and 2012 to 2016.

The photo‐protection offered by epidermal melanin is a possible factor explaining the differences in incidence between national groups.^10^ Although epidermal melanin content and phototype were not measured in the present study, Kazakh and Turkic/Asian people, in general, have more pigmented skin than Russian and European/Caucasian people. The incidence of BCC, SCC, and CMM in Astana is lower than that reported in European countries. For example, in Scotland, incidence rates in 2009 for CMM, BCC, and SCC in males were 17.3, 122.5, and 50.5, respectively, and in females were 20.3, 90.3, and 18.8, respectively.^1^ The incidence of non‐melanoma and melanoma skin cancers in Asian populations, such as in Singapore and Japan, is also lower than in predominantly Caucasian populations in Europe, USA, and Australia.^11^, ^12^, ^13^, ^14^ Apart from phototype, other factors which may differ between the population groups include personal photo‐protective measures, typical clothing, and outdoor/indoor occupations. These were not investigated in the present study.

As was found in Astana, BCC is the most common skin cancer in other Caucasian and Asian populations.^15^, ^16^ In Astana, the incidence of BCC was higher in Caucasian/European men than women. An increased incidence of BCC in males has previously been reported in European countries and may be due to higher sun exposure in men, who tend to have more outdoor work, expose a larger area of their skin, and are less likely to use sun protection than women.^1^, ^17^ Conversely, the incidence of BCC was higher in Kazakh and other Turkic/Asian females than in males, which may be due to differences in occupational or recreational exposures or sun protection practices or awareness. In agreement with other studies in Caucasian and Asian population groups, the head/neck was the most common site for BCC in both groups and sexes in Astana.^18^, ^19^

Similar to results in studies involving Caucasian, Chinese Asian, and Japanese populations, the second most common type of skin cancer in Astana was SCC.^1^, ^15^, ^16^, ^19^ The incidence of SCC in females was consistently higher than in males in Astana, which is the converse of that reported in several European countries.^1^, ^20^ The reasons for the higher incidence of SCC in women in Astana are not clear, but a study conducted in the Japanese population of Hawaii also demonstrated a higher incidence of BCC and SCC in females than males.^19^ The authors proposed that cultural and occupational differences between men and women may be responsible.

CMM was the least frequent skin cancer in Astana; the age adjusted incidences were slightly higher in men than women for both population groups. This is similar to reports of slightly higher incidences of CMM in men than women for Caucasian and Asian population groups.^15^, ^21^ Conversely, in Japan, it has been reported that malignant melanoma occurs more frequently in females than males.^15^ The most common body site for CMM in the Kazakh and other Turkic Asian group was the head and neck, whereas it was the trunk in the Caucasian/European group. However, when the population groups were divided with both sexes combined, no statistically significant association between national group and cancer site was found. The most frequent body site for CMM was the trunk for Russian males and the head and neck for Kazakh males, whilst it was the lower extremity for both Russian and Kazakh females. It has previously been reported that the predominant body site for CMM in Caucasian women and Caucasian men is the lower extremity and trunk, respectively.^22^ Interestingly, the sole of the foot was the commonest body sites for CMM in Japan, with acral lentiginous melanoma being the most frequent subtype.^23^ The difference in body site for CMM between Kazakh and Russian males in Astana may have been due to differences in the melanoma subtype; such information was not recorded in the cancer registry.

A limitation of our study is that it is based on a specialist oncology centre registry and not on a population‐based cancer registry. Therefore, the incidence of non‐melanoma skin cancer is likely to underestimate the true incidence, as tumours may be treated topically without histological verification, treated privately, or not treated at all. Differential use of health services over time and among different population groups may contribute to the rise in incidence rates and differences between groups. We could not assess how much of the rise in skin cancer incidence was due to changes in national composition, because population counts by age, sex, and national group were not available for the inter‐census years 2012 to 2016. We were also limited to using projected population estimates for 2014 which makes the analysis less reliable for this time‐period. A strength of the study is that it is one of the first to report skin cancer rates in a multi‐national transitional population during a period in which the population has rapidly expanded, partly due to an influx of Russian and other European groups.

The World Health Organisation has attributed the rising worldwide incidence of skin cancer to depletion of the ozone layer, and increasing outdoor activities and recreational exposures.^24^, ^25^ Thinning of the ozone layer has varied over the world, with most of it at polar latitudes: it has been insignificant over Kazakhstan, thus making ozone depletion as a local risk factor for skin cancer development unlikely.^26^ It is more likely that exposure to solar ultraviolet radiation (UVR) leading to sunburn and unprotected chronic exposures as a result of societal changes in behaviour have contributed to the increased risk of skin cancer found in the present study. Public health messages regarding protection of the skin from the damaging effects of UVR have yet to be adequately promoted in Kazakhstan [personal communication, Dr Khalel Imanbayev]. The implementation of educational sun protection programmes in schools and public forums in Kazakhstan is required to raise awareness of the carcinogenic effects of UVR exposure and hopefully to halt the steep rise in the incidence of skin cancer that has occurred over the past 10 years.

## FUNDING

This research did not receive any specific grant from funding agencies in the public, commercial, or not‐for‐profit sectors.

## CONFLICT OF INTEREST

None declared.

## AUTHOR CONTRIBUTIONS

Conceptualization: Pauline McLoone, Mary Norval, Khalel Imanbayev

Formal Analysis: Philip McLoone

Investigation: Khalel Imanbayev

Visualisation: Philip McLoone

Writing‐Original Draft Preparation: Pauline McLoone, Philip McLoone, Mary Norval

Writing‐Review and Editing: Mary Norval, Philip McLoone, Pauline McLoone, Khalel Imanbayev

## References

[1] deVries E , Arnold M , Altsitsiadis E , et al. Potential impact of interventions resulting in reduced exposure to ultraviolet (UV) radiation (UVA and UVB) on skin cancer incidence in four European countries, 2010‐2050. Br J Dermatol. 2012;167(Suppl 2):53‐62. 22881588 10.1111/j.1365-2133.2012.11087.x

[2] Apalla Z , Lallas A , Sotiriou E , Lazaridou E , Ioannides D . Epidemiological trends in skin cancer. Dermatol Pract Concept. 2017;7(2):1‐6. 10.5826/dpc.0702a01 PMC5424654 28515985

[3] Lomas A , Leonardi‐Bee J , Bath‐Hextall F . A systematic review of worldwide incidence of nonmelanoma skin cancer. Br J Dermatol. 2012;166(5):1069‐1080. 22251204 10.1111/j.1365-2133.2012.10830.x

[4] The agency on statistics of the Republic of Kazakhstan . Results of the 2009 national population census of the republic of Kazakhstan Analytical report. https://www.liportal.de/fileadmin/user_upload/oeffentlich/Kasachstan/40_gesellschaft/Kaz2009_Analytical_report.pdf; 2011 [accessed 15 July 2016].

[5] Ministry of national economy of the Republic of Kazakhstan, committee on statistics . Women and men in Kazakhstan, 2011–2015. http://stat.gov.kz/; 2016 [accessed 12 February 2017].

[6] Ministry of national economy of the Republic of Kazakhstan, committee on statistics. http://stat.gov.kz/ [accessed 16 February 2017].

[7] Weather 2 http://travel.com. http://www.weather2travel.com/january/kazakhstan/astana.php [accessed 10 October 2016].

[8] Cancer incidence in the Republic of Kazakhstan . Kazakh Research Institute of Oncology and Radiology 2015. Almaty. 2016;152.

[9] Eurostat . Revision of the European Standard Population—Report of Eurostat's task force 2013 [ONLINE] Available at: http://ec.europa.eu/eurostat/web/products-manuals-and-guidelines/-/KS-RA-13-028. [Accessed 18 April 2017].

[10] Tadokoro T , Kobayashi N , Zmudzka BZ , et al. UV‐induced DNA damage and melanin content in human skin differing in racial/ethnic origin. FASEB J. 2003;17(9):1177‐1179. 12692083 10.1096/fj.02-0865fje

[11] Lee HY , Chay WY , Tang MBY , Chio MTW , Tan SH . Melanoma: differences between Asian and Caucasian patients. Ann Acad Med Singapore. 2012;41:17‐20. 22499476

[12] Ichihashi M , Naruse K , Harada S , et al. Trends in non‐melanoma skin cancer in Japan. Recent Results Cancer Res. 1995;139:263‐273. 7597297 10.1007/978-3-642-78771-3_20

[13] Kim GK , Del Rosso JQ , Bellew S . Skin cancer in Asians. Part 1: non‐melanoma skin cancer. J Clin Aesthet Dermatol. 2009;2(8):39‐42. PMC2923966 20729955

[14] Bellew S , Del Rosso JQ , Kim GK . Skin cancer in Asians. Part 2: melanoma. J Clin Aesthet Dermatol. 2009;2(10):34‐36. 20725572 PMC2923934

[15] Gloster HM , Neal K . Skin cancer in skin of color. J Am Acad Dermatol. 2006;55(5):741‐760. 17052479 10.1016/j.jaad.2005.08.063

[16] Koh D , Wang H , Lee J , Chia KS , Lee HP , Goh CL . Basal cell carcinoma, squamous cell carcinoma and melanoma of the skin: analysis of the Singapore Cancer Registry data 1968‐97. Br J Dermatol. 2003;148(6):1161‐1166. 12828744 10.1046/j.1365-2133.2003.05223.x

[17] Oberyszyn TM . Non melanoma skin cancer: importance of gender, immunosuppressive status and Vitamin D. Cancer Lett. 2008;18:127‐136. 10.1016/j.canlet.2008.01.009 18267352

[18] Norval M , Kellet P , Wright CY . The incidence and body site of skin cancers in the population groups of South Africa. Photodermatol Photoimmunol Photomed. 2014;30(5):262‐265. 24417358 10.1111/phpp.12106

[19] Chuang TY , Reizner GT , Elpern DJ , Stone JL , Farmer ER . Non‐melanoma skin cancer in Japanese ethnic Hawaiians in Kauai, Hawaii: an incidence report. J Am Acad Dermatol. 1995;33(3):422‐426. 7657865 10.1016/0190-9622(95)91387-4

[20] Oshyvalova OO , Ziukov OL . Cutaneous squamous cell carcinoma: epidemiological aspects among the population of Ukraine. Wiad Lek. 2017;70:178‐181. 28511154

[21] Cress RD , Holly EA . Incidence of cutaneous melanoma among non‐hispanic whites, hispanics, asians and blacks: an analysis of California cancer registry data, 1988‐93. Cancer Causes Control. 1997;8(2):246‐252. 9134249 10.1023/a:1018432632528

[22] Leiter U , Garbe C . Epidemiology of melanoma and non‐melanoma skin cancer‐the role of sunlight. Adv Exp Med Biol. 2008;624:89‐103. 18348450 10.1007/978-0-387-77574-6_8

[23] Ishihara K , Saida T , Yamamoto A . Updated statistical data for malignant melanoma in Japan. Int J Clin Oncol. 2001;6(3):109‐116. 11706778 10.1007/pl00012091

[24] World Health Organisation . Ultraviolet radiation (UV). Skin cancers. http://www.who.int/uv/faq/skincancer/en/index1.html [accessed 13 January 2017].

[25] World Health Organisation . Ultraviolet radiation (UV). Sun protection. http://www.who.int/uv/sun_protection/en/ [accessed 14 September 2017].

[26] Bais AF , McKenzie RL , Bernhard G , et al. Ozone depletion and climate change: impacts on UV radiation. Photochem Photobiol Sci. 2015;14(1):19‐52. 25380284 10.1039/c4pp90032d

